# Analysis of Gene Expression Variance in Schizophrenia Using Structural Equation Modeling

**DOI:** 10.3389/fnmol.2018.00192

**Published:** 2018-06-11

**Authors:** Anna A. Igolkina, Chris Armoskus, Jeremy R. B. Newman, Oleg V. Evgrafov, Lauren M. McIntyre, Sergey V. Nuzhdin, Maria G. Samsonova

**Affiliations:** ^1^Institute of Applied Mathematics and Mechanics, Peter the Great St. Petersburg Polytechnic University, St. Petersburg, Russia; ^2^Zilkha Neurogenetic Institute, Keck School of Medicine, University of Southern California, Los Angeles, CA, United States; ^3^Department of Molecular Genetics & Microbiology, Genetics Institute, University of Florida, Gainesville, FL, United States; ^4^Department of Cell Biology, SUNY Downstate Medical Center, Brooklyn, NY, United States; ^5^Molecular and Computation Biology, University of Southern California, Los Angeles, CA, United States

**Keywords:** schizophrenia, neurodevelopmental disorders, structural equation models, signaling pathways, gene network modeling

## Abstract

Schizophrenia (SCZ) is a psychiatric disorder of unknown etiology. There is evidence suggesting that aberrations in neurodevelopment are a significant attribute of schizophrenia pathogenesis and progression. To identify biologically relevant molecular abnormalities affecting neurodevelopment in SCZ we used cultured neural progenitor cells derived from olfactory neuroepithelium (CNON cells). Here, we tested the hypothesis that variance in gene expression differs between individuals from SCZ and control groups. In CNON cells, variance in gene expression was significantly higher in SCZ samples in comparison with control samples. Variance in gene expression was enriched in five molecular pathways: serine biosynthesis, PI3K-Akt, MAPK, neurotrophin and focal adhesion. More than 14% of variance in disease status was explained within the logistic regression model (C-value = 0.70) by predictors accounting for gene expression in 69 genes from these five pathways. Structural equation modeling (SEM) was applied to explore how the structure of these five pathways was altered between SCZ patients and controls. Four out of five pathways showed differences in the estimated relationships among genes: between KRAS and NF1, and KRAS and SOS1 in the MAPK pathway; between PSPH and SHMT2 in serine biosynthesis; between AKT3 and TSC2 in the PI3K-Akt signaling pathway; and between CRK and RAPGEF1 in the focal adhesion pathway. Our analysis provides evidence that variance in gene expression is an important characteristic of SCZ, and SEM is a promising method for uncovering altered relationships between specific genes thus suggesting affected gene regulation associated with the disease. We identified altered gene-gene interactions in pathways enriched for genes with increased variance in expression in SCZ. These pathways and loci were previously implicated in SCZ, providing further support for the hypothesis that gene expression variance plays important role in the etiology of SCZ.

## Introduction

Schizophrenia (SCZ) is a psychiatric disorder with high heritability; estimates from twin and family-based studies suggest that the heritability of SCZ may be as high as 81% (Cardno and Gottesman, 2000; Sullivan et al., 2003; Lichtenstein et al., 2009; Wray and Gottesman, 2012). However, despite progress in identifying the genetic basis of the disorder, the molecular basis of SCZ remains elusive (Arslan, 2018). A recent GWAS of SCZ (the largest published), involving 36,989 SCZ cases and 113,075 controls, revealed 108 loci associated with the disease (Schizophrenia Working Group of the Psychiatric Genomics Consortium et al., 2014), and there are indications that the total number of loci associated with SCZ is much greater. However, while the estimated heritability of SCZ is high, the proportion explained by SNPs is smaller, with the genetic liability explained by SNPs estimated to be between 20%–35% when considering SNPs genome-wide (Lee et al., 2012; Cross-Disorder Group of the Psychiatric Genomics Consortium et al., 2013; Speed et al., 2017), or 3.4% when considering only SNPs with genome-wide significance in SCZ GWAS (Schizophrenia Working Group of the Psychiatric Genomics Consortium et al., 2014). The genetic component of SCZ is complex; rather than being explained by relatively few variants of large effect acting as fulcra of SCZ-associated molecular pathogenesis, the genetic susceptibility underlying SCZ may be a function of many variants contributing small effects which together dysregulate pathways and lead to SCZ. If SCZ is a disorder of pathways (Sullivan, 2012) GWAS alone is unlikely to offer immediate insight into the molecular basis of SCZ.

Studying gene expression profiles is a complimentary approach to GWAS and facilitates understanding the molecular etiology of SCZ. It allows the investigation of pathways and molecular networks affected by SCZ. The difficulties in studying gene expression in human patients with neurological disease has led to the adoption of relevant cellular models (Evgrafov et al., 2006) to facilitate understanding of cellular phenotypes. While post-mortem brain tissue samples are used to study gene expression changes in neurological diseases like SCZ (e.g., Chen et al., 2013; Lanz et al., 2015; Hu et al., 2016), these are comprised of terminally differentiated neurons and glial cells of an adult brain which has been subjected to many different environmental conditions. Because SCZ can be considered a neurodevelopmental disorder (Weinberger, 1987; Raedler et al., 1998; Lewis and Levitt, 2002; Alexander-Bloch et al., 2014), the use of port-mortem samples may not necessarily accurately capture the alterations in neurodevelopmental processes important in SCZ, but rather the consequences of these changes in terminally-differentiated cells. An alternative is to use cultured patient-derived neural progenitor cells, such as those derived from olfactory neuroepithelium (CNON; Wrobel et al., 2013), whereby neurodevelopmental changes can be modeled; environmental effects that are a component of post-mortem tissue samples can be reduced; and conditions can be standardized across samples. We analyzed the gene expression profiles in CNON cells from SCZ patients and control individuals to study alterations in gene expression reflective of the potential neurodevelopmental aspects of the disease. Recent analysis of this data identified genes differentially expressed in SCZ that are involved in Wnt and Notch signaling, and Serine biosynthesis pathways (Evgrafov et al., 2017).

Many of the studies on gene expression in diseases have focused on differences in mean expression between disease and non-disease groups. Analysis of the variance of gene expression both within genetically identical populations of cells or organisms and in genetically diverse populations is an emerging topic of discussion. It has provided insights in studies of various biological mechanisms, from evolution to embryonic development (Rönnegård and Valdar, 2011; Brown et al., 2014; Hoffman et al., 2014; Wang et al., 2014). There is evidence that suggests biological variance plays an important part in determining phenotypes (Ozbudak et al., 2002; Colman-Lerner et al., 2005; Raser and O’Shea, 2005; Cai et al., 2006; Manolio et al., 2009). Such variability, particularly in the context of gene expression, may be indicative of genomic or epigenomic influences on the function of a given gene or protein (Alemu et al., 2014). Genes with more constrained expression have been reported to be more likely to encode products with “housekeeping” functions, whereas genes with more variable expression tended to be those involved in developmental and environmental responses and more often associated with disease (Alemu et al., 2014). Interestingly, many of the SCZ-associated loci are also enriched for sequences that remained constant throughout primary evolution but evolved rapidly after the divergence of humans from chimpanzees (Pollard et al., 2006a). While these are predominantly noncoding sequences, they are thought to contain developmental gene regulatory elements and noncoding genes important in neurocognitive development (Pollard et al., 2006a,b; Hubisz and Pollard, 2014).

Although it has not been extensively studied, there is evidence that variance in biological processes is an important aspect of SCZ. For example, variation in cortical metabolic activity is elevated in SCZ patients compared to neurologically-healthy controls and bipolar disorder patients (Yang et al., 2014). In the context of gene expression, there have been two studies to date that have directly examined expression variance in SCZ (Mar et al., 2011; Zhang et al., 2016). In a study of patient-derived human olfactory neurosphere-derived (hONS) cells, several signaling pathways were found to have significantly altered mRNA expression variance in SCZ patient-derived hONS compared to control and Parkinson’s disease hONS (Mar et al., 2011). While high and low variance genes were observed in SCZ CNONs, the overall trend was a reduction in expression variance genome-wide (Mar et al., 2011). In a separate study, the variance in mRNA expression in peripheral blood mononuclear cells was found to be higher in early-onset SCZ patients compared to controls, and this variance was reduced after a 12-week treatment with oral antipsychotics (Zhang et al., 2016). These experiments highlight the apparent importance of biological variance in SCZ, however they examine different aspects of the disease, and the findings are somewhat difficult to reconcile. We therefore decided to examine gene expression variance in CNON cells between SCZ and controls in greater detail. We hypothesize that differences in expression variance between SCZ and control samples will enable us to detect SCZ-associated genetic perturbations (Mar et al., 2011; Mason et al., 2014; Zhang et al., 2016).

A variety of methods have been used to analyze the variance/covariance structure of gene expression profiles, e.g., differentially co-expression analysis (Watson, 2006; Lui et al., 2015), differential analysis of eigengene networks (Langfelder and Horvath, 2007), differential variability analysis (Ho et al., 2008; Jayaswal et al., 2013), PANA (Ponzoni et al., 2014), factor analysis (Coffman et al., 2005) and structural equation modeling (SEM). SEM is a multivariate statistical analysis technique based on Sewell Wright’s path analysis (Wright, 1918, 1921) and widely used in the fields of economics, psychology and sociology. SEM models a multiple-gene pathway structure by taking into account the direction of relationships among genes, allowing for complex interactions among genes where both mean and covariance structure of the data are modeled. In contrast, the coexpression analysis describes only relationships within a pair of genes. Network reconstruction methods which are based on partial correlations, describe chains of genes with related expression but do not account for covariances. SEM models can be built in an exploratory mode, but unlike other approaches, can also be deployed using an existing structure. As there are many already described gene networks (Kanehisa and Goto, 2000), SEMs provide a robust framework for modeling changes between environments in complex gene–gene interactions.

Recently, SEM was applied to analyze gene expression data and infer relationships between genes in gene regulatory networks (Li et al., 2006; Remington, 2009; Mi et al., 2010; Nock and Zhang, 2011; Fear et al., 2015). The attraction of SEM in this area resides in its ability to compare the path strengths between several nodes. The method was employed to predict perturbed gene interaction in neurological diseases, namely frontotemporal lobar degeneration with ubiquitinated inclusions, multiple sclerosis (Pepe and Grassi, 2014) and Parkinson’s disease (Pepe and Do, 2015). Moreover, SEM can be used to identify potential new gene interactions, as demonstrated in Fear et al. (2015). We use an SEM analysis of gene expression data in SCZ and controls to identify specific gene-gene relationships that differ in the context of a given pathway. Thus, in addition to identification of specific genes and implying involvement of corresponding pathways in SCZ, we anticipate identifying edges (gene-gene interactions) that are affected by SCZ in these pathways.

## Materials and Methods

### CNON Dataset

RNA-Seq transcriptome expression profiles of CNON cells from 144 SCZ patients (DSM-IV criteria) and 111 control individuals (no psychiatric disorders and no family history of SCZ) were used in this study. The details of biopsy and cell cultivation were described previously (Evgrafov et al., 2006; Wrobel et al., 2013). RNA libraries extracted from cells were prepared and sequenced on HiSeq2000 (Illumina) as previously described (Evgrafov et al., 2017). Reads were mapped to the combination of human genome, mtDNA and transcriptome, and those that were assigned to genes, were quantified by a custom RNA-Seq alignment pipeline, GT-FAR^1^. All the experimental and bioinformatics analysis steps were accompanied by quality control procedures; more detailed information is described in the previous study (Evgrafov et al., 2017). The data is available through dbGaP (Study ID: 26138). We carried out the analysis on 23,920 expressed genes (out of 59,902 total), normalized and filtered as in the previous study (Evgrafov et al., 2017).

### Selection of Candidate Pathways

In addition to pathways enriched in differentially expressed genes (Wnt, Notch, Serine biosynthesis; Evgrafov et al., 2017), we found that PI3K-Akt signaling has been identified as important in multiple studies of SCZ (Mao et al., 2009; Panaccione et al., 2013; Singh et al., 2013; Topol et al., 2015; Mulligan and Cheyette, 2016; Howell et al., 2017; Wang et al., 2017). Pathway analysis of GWAS data, performed by The Network and Pathway Analysis Subgroup of the Psychiatric Genomics Consortium (2015) showed enrichment of several GO terms, associated with neuron structure and histone H3-K4 methylation. Analysis of GWAS data (Chang et al., 2015) resulted in a large list of enriched pathways (insulin signaling pathway, neurotrophin signaling pathway, focal adhesion, VEGF signaling pathway, GnRH signaling pathway, tight junction and regulation of actin cytoskeleton) and likely candidate pathways directly connected to those enriched (Wnt signaling pathway, adherens junction, apoptosis, calcium signaling pathway, PI3K-Akt signaling pathway, leukocyte transendothelial migration, long-term potentiation, cell cycle and MAPK signaling pathway). Analysis of GWAS together with mutation and CNV data (Kotlar et al., 2015) revealed potential involvement of the ARC signaling complex, NMDAR complex, VGCC and FMRP target pathways, which play an important role in long-term potentiation and long-term depression pathway. These 19 KEGG pathways for which there is support for involvement with SCZ were the focus of our analysis.

### Variance in Gene Expression Between SCZ Patients and Control Individuals

We calculated the variances of gene expression for SCZ and control samples separately. Gene expression was estimated as in Evgrafov et al. (2017). We tested the null hypothesis that the number of genes with increased variance in the SCZ samples was equal or less than half of the total number of analyzed genes, using the sign test (Sheskin, 2006).

The 19 pathways were tested for enrichment for genes with increased variance in SCZ samples. We considered only genes annotated in the KEGG database. Using the variances calculated above, with a simple indicator for whether the variance was greater in SCZ, we applied an overrepresentation analysis (ORA) using the EASE score, a modified one-tail Fisher exact *p*-value implemented in DAVID (Hosack et al., 2003). We also applied a node-based ORA method (Gu et al., 2012). The node-based enrichment score is a sum of nodes containing at least one gene with increased variance. The null distribution of the enrichment score values was approximated by 1,000,000 simulation trials and the *p*-value of a pathway’s enrichment was calculated as the proportion of the trials having more extreme enrichments scores.

We also examined the residual variance after fitting age, sex, two principal components (PCs) and batch effect (Evgrafov et al., 2017). Residuals were calculated from DESeq2 tools (Love et al., 2014), and the null hypothesis that variance of the residuals in the SCZ group was equal to or less than that in the control group was tested using Levene’s test for each gene (Levene, 1960). We ranked the list of genes according to the estimated *p*-values, and applied the fast gene set enrichment analysis (FGSEA) method (available as the Bioconductor package “fgsea”; Sergushichev, 2016), for each pathway. We also conducted another Gene Set Analysis (GSA)–node-based enrichment analysis which aggregates *p*-values of Levene’s test by Fisher’s method (Leno-Colorado et al., 2017).

FDR-corrected *p*-values were calculated (Benjamini and Hochberg, 1995) and pathways significantly enriched for differential variance (FDR *p*-values < 0.01) were considered for further analysis.

### Logistic Regression Analysis

To determine whether the genes in pathways enriched for increased variance were also associated with disease status, we estimated the overall correlational structure among these genes. Residuals from the DEseq2 model (described above) for all the genes in these enriched pathways were used in a principle component analysis. PC’s which explained at least 1% of the variance among the genes were used as dependent variables in a logistic regression with SCZ status as the outcome. To assess the model fit we use the C-index (i.e., area under receiver operating characteristic curve, ROC) as a measure of goodness-of-fit for binary outcomes in the model. A value of 0.5 means that the model is no better in predicting an outcome than random chance (Freedman, 2009).

### SEM Analysis

SEM is a statistical technique that is first proposed by S. Wright as path analysis (Wright, 1918, 1921). Modern SEM models contain two distinct parts: structural and measurement models (Bollen, 1989; Kline, 2011). Structural models reflect the causal dependencies between endogenous and exogenous variables as the following system of linear equations:

$$\left[\begin{array}{c} y\\ \eta \end{array}\right]=\text{B}\left[\begin{array}{c} y\\ \eta \end{array}\right]+\Gamma \left[\begin{array}{c} x\\ \xi \end{array}\right]+\zeta ,$$

where *x* and *y* denote vectors of observed variables (exogenous and endogenous, respectively), *ξ* and *η* are vectors of latent variables (exogenous and endogenous, respectively), *B* is a matrix of coefficients linking endogenous variables, Г is a matrix of coefficients relating exogenous variables to endogenous, and *ζ* is a vector of structural errors. The measurement model describes the relations between latent variables and their indicators:

$$z=\Lambda \left[\begin{array}{c} \eta \\ \xi \end{array}\right]+\delta ,$$

where *z* denotes a vector of observed indicators, Λ is a matrix of factor loadings of the indicators *z* of a latent variables *η* and *ξ*, and *δ* is a vector of measurement errors. SEM makes the following assumptions: the variable *ξ* in uncorrelated with the error *ζ*, the variable $\left[\begin{array}{c} \eta \\ \xi \end{array}\right]$ is uncorrelated with the error *δ*, the error *ζ* is uncorrelated with the error *δ*, matrix *I*–*B* is nonsingular (where *I* is the identity matrix). In addition, the covariance matrixes for *ξ*, *δ* and *ζ* are known. Considering the structural model and the assumptions, the model-implied covariance matrix between observed variables, Σ is expressed as a function of parameter matrices (Bollen, 1989). Parameter estimation is performed by minimizing the difference between the model-implied covariance matrix Σ and the observed sample covariance matrix *S* by the Maximum Likelihood method (Kline, 2011). Here we considered SEM without exogenous latent variables. We also set covariance between each pair of exogenous variables to zero. In order to fix the scale of latent variables we put their variance to 1 and mean to 0 (Reference-Group Method; Little et al., 2006). Potential non-linear interactions and quadratic effects were not included in the modeling.

To compare SEM estimates between different subgroups of the data (SCZ and control samples, here) the multiple group (multigroup) modeling is applied (Pugesek et al., 2003). This technique simultaneously assesses parameters of multiple models and can examine the null hypothesis whether the two parameters are equal (Rosseel, 2012). Here we examined the differences between all structural parameters in SCZ and control groups. The multigroup modeling also allows us to compare parameters between groups. We considered five covariates—age, sex, PCs and batch effects. For each observed variable we constructed a linear equation where the covariates played the role of explanatory variables. We added these equations to the model and constrained equation parameters to be equal between groups. The multigroup SEM analysis was performed with lavaan tools (Rosseel, 2012). Two scores—Root mean square error of approximation (RMSEA) and comparative fit index (CFI)–were used to select the model (Hermida et al., 2015). RMSEA is a measure of the difference between the fit model and the data. Low values indicate models that describe the data well. CFI varies from zero (the proposed model fits no better than the baseline model) to one—values closer to one indicating a better model fit. The best model was defined as the one with the highest CFI value within models with RMSEA < 0.2. We also calculated the Akaike information criterion (AIC) and Bayesian information criterion (BIC) values for each model in the multigroup analysis to assess which group of samples (SCZ or control) is better explained by the model.

Visualization of path diagrams for SEM models was performed with DiagrammeR, an R package^2^.

### Construction of SEM Models

Gene networks of interest were constructed based on the KEGG database and literature data (Supplementary Figure S1). These networks contained several nodes with more than one gene. Such a situation may arise when the node genes encode protein complex subunits or they are members of a multi-gene family. Moreover, it may happen that a gene encodes several isoforms of one protein, and either the exact isoform involved is unknown, or the isoform varies depending on cellular environment (Luo and Brouwer, 2013; Luo, 2017).

To address the precise configuration of a network, we generated a collection of structural equation models corresponding to all possible alternative networks where each complex node was substituted with one of the constituent genes. Parameter estimation was performed for each model by multi-group SEM analysis (Rosseel, 2012). If during the estimation a model became empirically non-identifiable, it was discarded. Within the identifiable model set we selected the models that had a low number of non-significant path coefficients (less or equal to 3) on the control set of samples and low RMSEA value (less than 0.2). A model with the highest value of CFI within the remaining models was defined as the best one and reflected the precise configuration of the network. The runtime of the pipeline to select the best model linearly depends on the number of alternative models.

## Results

### Analysis of Heterogeneity of Gene Expression Variance Between SCZ and Control Samples

We analyzed variance in gene expression profiles of CNON cells of 144 SCZ patients and 111 control individuals with no psychiatric disorders or family history of SCZ. All the experimental and bioinformatics pre-processing steps are previously described (Evgrafov et al., 2017). We analyzed 23,920 expressed genes and tested the null hypothesis that variance of the SCZ group was less than or equal to the control groups. We found significantly more genes with increased variance in the SCZ group (sign test, *p*-value < 0.01). In total, 16,434 genes had an increased variance amongst the SCZ group, and 7486 genes had a higher variance amongst controls (S1 Supporting Information).

Two Over Representation Analyses (ORA) and two Gene Set Analyses (GSA) were applied to 19 pathways (Table 1). Five pathways showed a higher than expected (FDR *p*-value < 0.01) number of genes with increased variance in the SCZ group according to the node-based ORA (Table 1). In addition, two pathways were significantly enriched for genes with increased expression variance using node-based GSA method (based on the Levene’s test and Fisher’s method) on the residuals (FDR *p*-values < 0.01; Table 1). These two sets were merged into one set of five unique pathways for further analysis—serine biosynthesis, PI3K-Akt, MAPK, neurotrophin signaling and focal adhesion (Table 1).

**Table 1:** The enrichment of signaling pathways with genes that have increased variances in Schizophrenia (SCZ) samples.

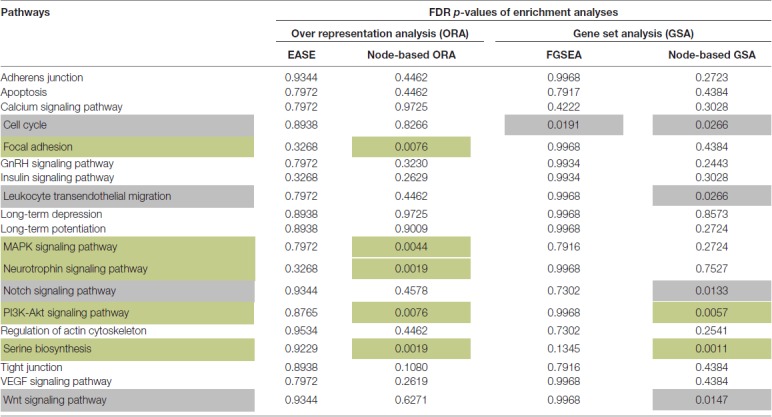

*FDR p-values of four enrichment analyses (two ORA and two GSA) is presented. Green color marks pathways, which were significantly enriched (FDR p-value < 0.01) for genes with increased expression variance in at least one analysis. Gray color marks pathways, which were not significantly enriched, but demonstrated FDR p-values < 0.05*.

### The Logistic Regression Fit for Disease Status

Serine biosynthesis, PI3K-Akt, MAPK, neurotrophin and focal adhesion signaling pathways include 69 unique genes; several of these genes are shared between these five pathways. PC analysis on residuals for 69 genes identified 15 PCs which individually explained at least 14% of variance. The logistic regression fit for disease status (SCZ vs. control) with these 15 PCs as factors revealed three PCs with *p*-value < 0.05 and six PCs with *p*-value < 0.1. The C-index of the fit was equal to 0.70 indicating that variance among genes in these five pathways is associated with disease status in individuals (coefficient of determination, *R*^2^ = 0.14).

### Identification of Important Regulatory Relationships Using SEM

Five pathways enriched for genes with increased expression variance in SCZ samples (serine biosynthesis, PI3K-Akt, MAPK, neurotrophin signaling and focal adhesion pathways) were modeled with SEM. Under model identification constraints (Supplementary Text S1) the number of nodes in a network for modeling was limited to 14 nodes, and therefore we reduced the five KEGG pathways to only the key interactions in these pathways (Table 2). The reduced networks of Serine biosynthesis, PI3K-Akt, MAPK, neurotrophin signaling and focal adhesion pathways were denoted as initial, consisted of 5, 11, 11, 9 and 11 nodes and included 5, 17, 20, 11 and 27 genes, respectively (see Table 2 and Supplementary Figure S1). The number of genes was higher that the number of nodes as several nodes were complex (i.e., contained more than one-member gene).

**Table 2:** List of genes included in the initial networks.

Pathway	Genes	Number of genes	Number of nodes
Serine biosynthesis	*PHGDH, PSAT1, PSPH, SHMT1, SHMT2*	5	5
PI3K-AKT	*IRS1, PIK3CA, PIK3CB, PIK3CD, PIK3R1, PIK3R2, PIK3R3, PTK2, PDPK1, PTEN, AKT3, nCRTC2, FOXO3, BAD, GSK3B, TSC1, TSC2*	17	11
MAPK	*SOS1, SOS2, MRAS, HRAS, KRAS, NRAS, RASA2, NF1, RAPGEF2, PRKCA, PRKCB, PRKCG, RAF1, BRAF, MAP2K1, MAP2K2, MAPK1, MAPK3, RAP1A, RAP1B*	20	11
Neurotrophin	*NGFR, TRAF6, RAC1, MAP3K1, MAP2K7, MAP8, MAP9, MAP10, TP73, TP53, JUN*	11	9
Focal adhesion	*PTK2, BCAR1, CRKL, CRK, DOCK1, PIK3CA, PIK3CB, PIK3CD, PIK3R1, PIK3R2, PIK3R3, VAV2, VAV1, VAV3, RAC1, RAC2, RAC3, PAK1, PAK2, PAK3, PAK4, PAK5, PAK6, RAPGEF1, RAP1B, RAP1A, PTEN*	27	11

We generated alternative structural equation models substituting complex nodes within initial networks with each member gene. One, 12, 192, 3 and 1296 alternative models for serine biosynthesis, PI3K-Akt, MAPK, neurotrophin signaling and focal adhesion signaling pathways were generated, respectively. The multigroup SEM fit was then performed for each alternative model, and the best model for each pathway was identified using three characteristics: number of non-significant path coefficients, RMSEA and CFI. Models that were empirically non-identifiable or contained more than three non-significant path coefficients in the estimation on control set or showed RMSEA values more than 0.2 were discarded. The best model for each pathway was selected by the highest CFI index value among the models with RMSEA 0.2 or less. For each of the five pathways the values of both AIC and BIC criteria were higher in model fits for SCZ samples compared to controls indicating that the gene expression data in control group of samples were better explained by the structures of pathways.

**Figure 1:**
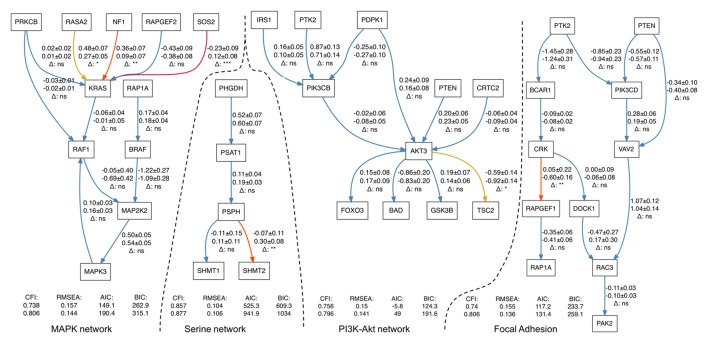
Structural equation modeling (SEM) fits for four gene networks representing serine biosynthesis, PI3K-Akt, MAPK and focal adhesion signaling. Each arrow contains three-line text information: the first line is the estimation of a path coefficient on control set of samples (and the standard error); the second line is the estimation of a path coefficient on Schizophrenia (SCZ) set; the third line shows the significance of difference between the estimates. *p*-values higher than 0.05 are marked by “ns” and blue color (non-significant), less than 0.05–by (*) and yellow color less than 0.01–by (**) and red color less than 0.001–by (***) and dark red color.

In four out of the five pathways, we found statistically significant differences in path coefficients (*p*-value < 0.05; Table 3). These coefficients reflect the strength of causal interactions between *NF1*, *SOS2* and *RASA2* with *KRAS* in MAPK pathway; between *PSPH* and *SHMT2* in serine biosynthesis; between *AKT3* and *TSC2* in PI3K-Akt signaling pathway; and between *CRK* and *RAPGEF1* in the focal adhesion pathways (Figure 1). SEM fit of neuroptrophin signaling pathway is shown in Supplementary Figure S2. The path coefficient represents the relationship between two genes, in the context of the entire pathway. A significant difference in a path coefficient between the two environments indicates that the relationship between the two genes is altered in the disease compared to the non-disease. All calculations were implemented in R. The link to the GitHub repository containing R scripts is provided in the Supplementary Material.

**Table 3:** The gene interactions with statistically significant differences in path coefficients between SCZ and control samples.

Interaction	Pathway	Significance
*PSPH → SHMT2*	Serine biosynthesis	*p*-value < 0.01
*SOS1 → KRAS*	MAPK signaling	*p*-value < 0.001
*NF1 → KRAS*	MAPK signaling	*p*-value < 0.01
*RASA2 → KRAS*	MAPK signaling	*p*-value < 0.05
*AKT3 → TSC2*	PI3K-Akt signaling	*p*-value < 0.05
*CRK → RAPGEF1*	Focal adhesion	*p*-value < 0.01

## Discussion

SCZ is highly heritable severe mental disorder with clinically heterogeneous symptoms (Picardi et al., 2012; Takahashi, 2013) and different risk factors (Mäki et al., 2005; Serafini et al., 2012). Several lines of evidence point to neurodevelopment as playing a primary role in etiology of the disease (Weinberger, 1987; Raedler et al., 1998; Lewis and Levitt, 2002). To elucidate neurodevelopmental aspects of SCZ mechanisms we studied gene expression profiles in CNON cells derived from SCZ patients and healthy individuals, a suitable experimental model to analyze the neurodevelopment processes (Evgrafov et al., 2006, 2017; Wrobel et al., 2013). SCZ-related genes that differ between SCZ and control groups in the mean expression levels have been identified from these data (Evgrafov et al., 2017). Here, we hypothesized that variance in gene expression is altered in SCZ CNON cells and found that SCZ CNON cells showed higher variance in expression than the control cells.

Of the 19 pathways related to neurodevelopment and associated with SCZ-associated tested, five pathways (serine biosynthesis, PI3K-Akt, MAPK, neurotrophin and focal adhesion signaling pathways) were enriched for genes with increased variance among the SCZ samples. There were 69 genes in these five pathways. Residuals from individual models that account for covariates were used to estimate the covariance among these genes in a PCs model. PC’s explained 14% of the variance in disease status, suggesting that the co-variance in expression among these genes and pathways are potential predictors of SCZ. Several edges (gene-gene interactions) are altered: *NF1*, *SOS2* and *RASA2* with *KRAS* in MAPK pathway; between *PSPH* and *SHMT2* in serine biosynthesis; between *AKT3* and *TSC2* in PI3K-Akt signaling pathway; and between *CRK* and *RAPGEF1* in the focal adhesion pathways.

Previous examination of gene expression variance in SCZ has been somewhat contradictory. In an earlier study involving patient-derived hONS cells (which resemble CNON cells), variance in gene expression was found to be more constrained genome-wide in SCZ cases than in controls or Parkinson’s disease (Mar et al., 2011), whereas in blood, expression variance tended to be higher in SCZ than in controls (Zhang et al., 2016). It should be noted that both of these studies had relatively small sample sizes, and therefore may not have captured the entire breadth of expression variance in SCZ. Similarly, in the earlier CNON study, although the authors did not analyze variance in expression of all genes, it was noted that there were genes with high expression variance in SCZs in some of the same pathways that we examined in this study (Evgrafov et al., 2017).

There is support in the literature for the interactions highlighted in our study in SCZ and, more broadly, other neurodevelopmental disorders. Interactions of both NF1 and SOS1 genes with NRAS (homolog of KRAS) gene in MAPK pathway have been previously associated with Noonan syndrome (Baralle et al., 2003; Longoni et al., 2010), a multisystem developmental disorder that includes perturbed neurodevelopment (Noonan, 2005; Roelofs et al., 2016), and RASA2 mutations have also been reported in individuals with Noonan syndrome (Chen et al., 2014). While Noonan syndrome has a large array of physiological abnormalities, and that we did not explicitly examine interactions with NRAS (having collapsed the corresponding, RAS family node of the MAPK pathway to KRAS), this suggests that changes in how NF1 and SOS1 interact with the RAS genes contribute towards neurodevelopmental disorders (like SCZ and Noonan syndrome), possibly through altered coordination of signaling to the RAS/RAF component of the MAPK pathway. Under normal conditions, SOS1 activates RAS proteins (including KRAS) by removal of bound GDP from RAS, thus freeing RAS to bind GTP and activate downstream components of MAPK signaling (Boriack-Sjodin et al., 1998). In contrast, NF1 inhibits RAS activity by hydrolyzing bound GTP (Bollag et al., 1996; Corral et al., 2003), and RASA2 also suppresses RAS activation by enhancing RAS’s GTPase activity (Gaul et al., 1992; Maekawa et al., 1994; Arafeh et al., 2015). In our analysis, we found that the path coefficients the RASA2–KRAS and NF1–KRAS interactions were smaller in SZ than in controls, potentially indicating that the relationship between these genes in terms of gene expression is weaker in SCZ, particularly for the NF1–KRAS which shows a large difference between SCZ and controls. However, the most pronounced change is in the SOS1-KRAS interaction, where the path coefficient changes direction indicating a dramatic shift in the gene expression relationship between these two genes. The alterations in SOS1–KRAS, NF1–KRAS and RASA2–KRAS interactions reflect transcriptional dysregulation of MAPK signaling, centered on a specific mechanism (i.e., RAS activation/inhibition). RAS activation is a tightly-regulated process, and its overactivation leads to oncogenesis and various developmental syndromes (Bos, 1989; Rajalingam et al., 2007; Tidyman and Rauen, 2009). It is possible that the changes in how the expression of these genes relate to one another might explain the differences we observed in our analysis of the MAPK pathway, by perturbing the regulation of RAS activation/inhibition. We also note that paths downstream of KRAS are not significantly different between SCZ and controls, indicating that the downstream components of the MAPK pathway are unperturbed in SCZ. This taken with regulation of RAS activation by SOS1, NR1 and RASAS2 suggests that dysregulation RAS activation may contribute towards SCZ risk. This could also potentially explain abnormal MAPK activity previously reported in SCZ. SCZ-associated abnormalities in regulation of RAS/MEK/ERK pathway have been reported upstream of RAS family proteins, as the product of the SCZ risk gene, Disrupted in SCZ 1 (DISC1), interacts with RASSF7 in the brain (Wang et al., 2016), consistent with our findings. Other components of the MAPK signaling pathway such as RAS-related guanine exchange factors are also implicated in SCZ risk (Xu et al., 2008, 2009; Levy et al., 2015). These guanine exchange factors activate RAS by exchanging bound GDP for free GTP (Rebhun et al., 2000; Feig, 2011) and are important in the development of cortical neurons and regulation of neuronal function (Maeta et al., 2016). Taken all together and given that SOS1, NR1 and RASA2 regulate RAS activation by controlling its GTPase activity, this highlights the dysregulation of RAS activation as a possible mechanism in SCZ development.

In serine biosynthesis we found one interaction (between *PSPH* and *SHMT2*) altered between SCZ and controls. In the previous DE analysis, *PSPH* gene showed tendency to be more highly expressed in SCZ compared to controls, while another member of the same pathway, *PSAT1*, was a differentially expressed gene after FDR correction (Evgrafov et al., 2017). Moreover, samples with the lowest expression level of these genes were also from SCZ group. The observation that the highest and lowest expression levels of these genes were observed among SCZ samples in this study supports our hypothesis of increased gene expression variance in SCZ. Mutations in *PSAT1*, which regulates *PSPH*, affect neurodevelopment (Tabatabaie et al., 2010). There is also evidence that the gene is implicated in SCZ based on a study of gene expression in a family with a chromosomal translocation near *PSAT1* associated with SCZ and schizotypal personality disorder (Ozekia et al., 2012). The second gene in the interaction, *SHMT2*, demonstrated the overexpression and regulatory function (suppression and promotion) in migration and proliferation in carcinoma cells (Wu et al., 2016).

The altered interaction in the PI3K-Akt signaling pathways identified in the current study involves *AKT3*, an excellent SCZ candidate gene. GWAS studies identified several SNPs with genome-wide significance inside or in the vicinity of this gene, suggesting that *AKT3* is a good positional candidate. *AKT3* encodes for serine/threonine kinase, a regulator of cell signaling in response to insulin and growth factors. Such a central position in an interrelated network of intracellular signaling implies a role in many cellular and organismal processes, including development. Multiple functional and genetic studies (Howell et al., 2017) clearly indicate the important role of this gene in brain development and suggest involvement in SCZ, and deletion of the gene in mice results in a phenotype reminiscent of a psychiatric manifestation (Bergeron et al., 2017). In addition, the *RP11–370K11.1* gene, one of the differentially-expressed genes identified in the same dataset (Evgrafov et al., 2017), is located within *AKT3* gene, and one of the SNPs strongly associated with SCZ resides within *RP11–370K11.1*. Another component of the interaction found to be altered between SCZ and controls is *TSC2*, and genetic variation in the *TSC2* locus is associated with SCZ risk (The International Schizophrenia Consortium et al., 2009).

Finally, the focal adhesion pathways contained one interaction (between *CRK* and *RAPGEF1* genes) that has a significantly different path coefficient between SCZ and control SEM models. Both *CRK* and *RAPGEF1* genes are included in the SZGR2.0 database (Jia et al., 2017) as related to SCZ risk. Remarkably, dynamics of focal adhesion is altered in neural stem/progenitor cell cultures derived from olfactory neuroepithelium of SCZ patients (Féron et al., 1999; Fan et al., 2013), a cellular model similar to CNON cells. Focal adhesion controls motility (Mitra et al., 2005) is also affected in neural stem/progenitor cells derived from SCZ patients (Tee et al., 2017). Involvement of focal adhesion in etiology of SCZ is plausible if not expected as it mechanistically explains aberrations in brain development through affected control of proper migration of neural progenitor cells.

As expected, our variance-based analysis results are overall consistent with the differential gene expression (DEX) analysis of mean expression (Evgrafov et al., 2017), and identification of serine biosynthesis pathways in both analyses is a clear example. However, there are also substantial differences. First, in the current study, we identified five pathways disturbed in SCZ, while simple overrepresentation test of DEX results does not return any significant KEGG pathways. Second, SEM method is specifically designed to identify disturbed relationships between genes, and, in wider terms, affected pathways and networks. Thus, SEM could be especially helpful in deciphering molecular etiology of the disease. As we found, both approaches can identify the same genes, when, for example, the increased variance is explained mostly by a difference in mean between groups of samples. However, there are certainly cases when mean is not changed significantly, while SCZ samples are characterized by higher gene expression. We consider these approaches as mostly complementary and suggest that a combination of both methods should be used to find differences in cell regulation associated with SCZ.

In summary, our analysis provides evidence that variance and covariance in gene expression is an important characteristic of SCZ etiology. The application of SEM identified five interactions between genes in serine biosynthesis, PI3K-Akt, MAPK and focal adhesion pathways that were altered in SCZ neural progenitor cells, suggesting that these interactions are important in the molecular etiology of SCZ. The association of these interactions with SCZ is well supported by literature data that allows us to consider them as good candidates for further downstream analysis.

## Author Contributions

AI, LM, SN and MS: conceptualization and methodology. OE and CA: data curation. AI: formal analysis and visualization. AI, JN, OE, LM, SN and MS: investigation. SN and MS: project administration. OE: resources. MS and LM: supervision. AI and MS: writing–original draft. AI, CA, JN, OE, LM, SN and MS: writing–review and editing.

## Conflict of Interest Statement

The authors declare that the research was conducted in the absence of any commercial or financial relationships that could be construed as a potential conflict of interest.

## Footnotes

^1^https://genomics.isi.edu/gtfar

^2^http://rich-iannone.github.io/DiagrammeR/

## Supplementary Material

The Supplementary Material for this article can be found online at: https://www.frontiersin.org/articles/10.3389/fnmol.2018. 00192/full#supplementary-material
