# Supplementary material for: Analysis of Gene Expression Variance in Schizophrenia Using Structural Equation Modeling
Source: Front Mol Neurosci. 2018 Jun 11;11:192. doi: 10.3389/fnmol.2018.00192 (PMC6004421; doi:10.3389/fnmol.2018.00192)
Supplement: Supplementary file 2 [file Presentation_1.PDF]

Supplementary Material

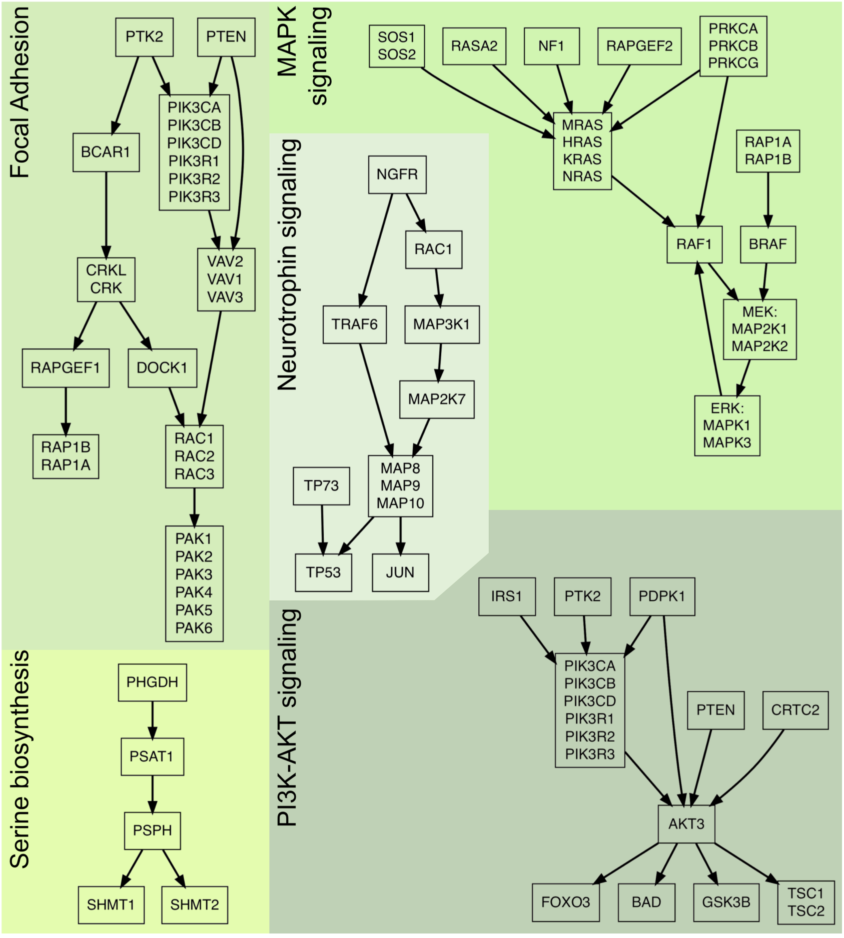

Fig. S1. Initial configurations of five gene networks: serine biosynthesis, PI3K-Akt, MAPK, neurotrophin and focal adhesion. Rectangles with several lines represent complex nodes with alternative member genes.

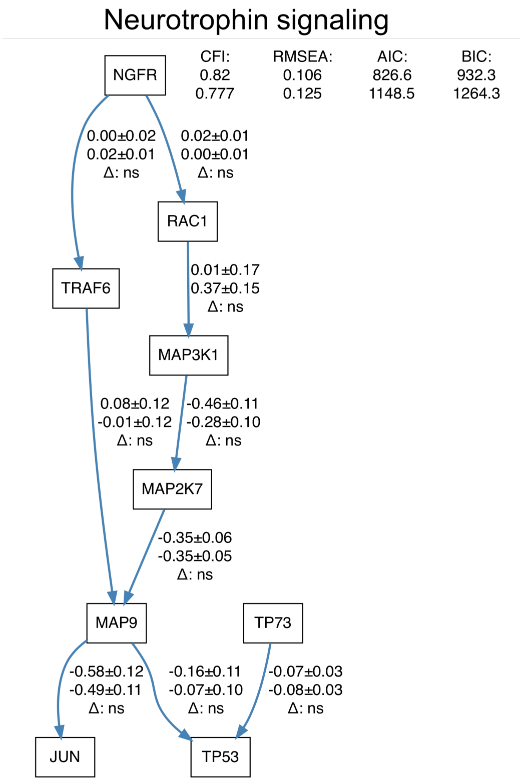

Fig. S2. SEM fit of Neurotrophin signaling pathway. Each arrow contains three-line text information: the first line is the estimation of a path coefficient on control set of samples (and the standard error); the second line is the estimation of a path coefficient on SCZ set; the third line shows the significance of difference between the estimates. p-values higher than 0.05 are marked by “ns” and blue color (non-significant), less than 0.05 - by (*) and yellow color, less than 0.01 – by (**) and red color, less than 0.001 – by (***) and dark red color.

Text S1

Model identification

All the models were tested for the Rank and Order conditions that are necessary and sufficient for identification of structural models (Gujarati, 2004). Let N be a total number of variables (exogenous and endogenous), M be the number of equations and K be the number of exogenous variables in a model, m_i be the number of variables within ith equation, and k_i be the number of exogenous variables within ith equation. Then the first and the second order conditions are formulated as follows:

$$N \geq M - 1$$
$$K - k_i \geq m_i - 1$$

In order to check the rank condition, the structural equation is transformed into binary matrix form. To test the rank condition of ith equation the following steps are performed: (1) remove all rows from the matrix where ith column has ones, (2) remove ith column, (3) calculate the matrix rank. If the rank is equal to (M-1) than ith equation is identified. In addition to the formal identification rules there is an empirical step that is essential during the parameter optimization procedure (Kenny, 1979). This involves examining the parameter estimates to ensure they are all within logical bounds. For example, variances that are estimated as negative should be zero.

The number of data samples limits the number of network nodes. The number of independent values in the covariance matrix of observed variables should be less than the number of samples. In the current project the number of observed genes in a network denoted by p should satisfy the following constraint $p(p+1)/2 \leq \min(111, 144)$, $p \in Z_{+}$, where 111 and 144 represent the number of control and SCZ samples, respectively. This constrains the number of nodes in the network to 14 or less.

References

Gujarati, D. N. (2004). *Basic Econometrics (Fourth Edition)*. The McGraw–Hill Companies.
Kenny, D. A. (1979). *Correlation and causality*. Wiley.

Repository with the code

https://github.com/iganna/sem_config
